# Predicting the Survival and Immune Landscape of Colorectal Cancer Patients Using an Immune-Related lncRNA Pair Model

**DOI:** 10.3389/fgene.2021.690530

**Published:** 2021-09-06

**Authors:** Chao Ma, Xin Zhang, Xudong Zhao, Nan Zhang, Sixin Zhou, Yonghui Zhang, Peiyu Li

**Affiliations:** ^1^Medical School of Chinese PLA, Beijing, China; ^2^Department of General Surgery, The First Medical Center, Chinese PLA General Hospital, Beijing, China; ^3^State Key Laboratory of Proteomics Beijing Proteome Research Center National Center for Protein Sciences (Beijing), Beijing Institute of Lifeomics, Beijing, China

**Keywords:** colorectal cancer, prognosis, immune-related lncRNA pairs, immunotherapy, tumor-infiltrating immune cells

## Abstract

**Background:**

Accumulating evidence has demonstrated that immune-related long non-coding ribonucleic acids (irlncRNAs) can be used as prognostic indicators of overall survival (OS) in patients with colorectal cancer (CRC). Our aim in this research, therefore, was to construct a risk model using irlncRNA pairs with no requirement for a specific expression level, in hope of reliably predicting the prognosis and immune landscape of CRC patients.

**Methods:**

Clinical and transcriptome profiling data of CRC patients downloaded from the Cancer Genome Atlas (TCGA) database were analyzed to identify differentially expressed (DE) irlncRNAs. The irlncRNA pairs significantly correlated with the prognosis of patients were screened out by univariable Cox regression analysis and a prognostic model was constructed by Lasso and multivariate Cox regression analyses. A receiver operating characteristic (ROC) curve was then plotted, with the area under the curve calculated to confirm the reliability of the model. Based on the optimal cutoff value, CRC patients in the high- or low-risk groups were distinguished, laying the ground for evaluating the risk model from the following perspectives: survival, clinicopathological traits, tumor-infiltrating immune cells (TIICs), antitumor drug efficacy, kinase inhibitor efficacy, and molecules related to immune checkpoints.

**Results:**

A prognostic model consisting of 15 irlncRNA pairs was constructed, which was found to have a high correlation with patient prognosis in a cohort from the TCGA (*p* < 0.001, HR = 1.089, 95% CI [1.067–1.112]). According to both univariate and multivariate Cox analyses, this model could be used as an independent prognostic indicator in the TCGA cohort (*p* < 0.001). Effective differentiation between high- and low-risk patients was also accomplished, on the basis of aggressive clinicopathological characteristics, sensitivity to antitumor drugs, and kinase inhibitors, the tumor immune infiltration status, and the expression levels of specific molecules related to immune checkpoints.

**Conclusion:**

The prognostic model established with irlncRNA pairs is a promising indicator for prognosis prediction in CRC patients.

## Introduction

Colorectal cancer (CRC), also named as bowel cancer, is a form of malignancy that affects the large intestine. Individuals diagnosed with CRC may have a huge variety of clinical symptoms, ranging from intestinal bleeding, changes in bowel habits, to anemia or abdomina pain (Dekker et al., 2019). As shown in the GLOBOCAN 2020 estimates released by the International Agency for Research on Cancer, there were approximately 1.9 million new CRC (including anus) cases and 935,000 CRC-related deaths worldwide. Of all cancer types, CRC ranked third in incidence but second in mortality (Sung et al., 2021). According to etiology studies, risk factors having strong associations with disease incidence include male, older age (Bailey et al., 2015; Wolf et al., 2018), heredity factors (Jiao et al., 2014), smoking (Botteri et al., 2008), excessive alcohol intake (Cai et al., 2014), body weight (Kyrgiou et al., 2017), and red or processed meat intake (Chan et al., 2011). However, these risk factors do not offer information on the biological behavior of cancer cells and thus do not possess precise predictive value for oncological outcomes. Although the advancement of primary and adjuvant treatment techniques has dramatically enhanced the overall survival (OS) of patients in the advanced stage, prognosis remain best for those in the early stage (Van Cutsem et al., 2016; Ciardiello et al., 2019; De Falco et al., 2020; Morse et al., 2020). Therefore, identification of novel prognostic biomarkers, a comprehensive understanding of the molecular mechanisms and the construction of a more precise model for predicting patient prognosis are clearly essential and urgently required.

The tumor microenvironment (TME), the environment surrounding a tumor, is composed of the extracellular matrix, chemokines, cytokines, and non-tumor cells (Chen et al., 2015). The tumors and their TME constantly interact with and affect each other. On the one hand, tumors can produce extracellular signals, promote tumor angiogenesis, and induce peripheral immune tolerance, thereby affecting the TME. On the other hand, the immune cells in the TME can inhibit tumor growth by attacking and removing tumor cells through immune response (Pedrosa et al., 2019). The recent years have seen an increasing number of models incorporating long non-coding ribonucleic acids (lncRNAs), the non-protein-coding RNAs with lengths beyond 200 nucleotides, as they have predictive and prognostic value in CRC diagnosis and treatment (Denaro et al., 2019; Poursheikhani et al., 2021). Immune-related lncRNAs (irlncRNAs) may direct the expression of genes associated with immune cell activation, leading to the infiltration of tumor immune cells. They not only alter the genome or transcriptome but also modify the immune microenvironment, making them conducive to the malignant phenotypes of cancer (Heward and Lindsay, 2014). These irIncRNAs can be effectively incorporated into tumor immune infiltration models. Mu et al. (2020) identified 14 irlncRNAs and established a model for CRC prognosis prediction. Xu J. et al., 2019 revealed the oncogenic role of the irlncRNA MIR17HG in CRC and its potential as a new target for treatment. Li et al. (2020) explored a signature based on seven immune-related lncRNAs for predicting the OS of patients with colon adenocarcinoma (COAD). Lin et al. (2020) constructed a signature with nine irlncRNAs and verified its clinical value in predicting prognosis and guiding tailored therapy for colon cancer patients.

However, sampling bias and technical batch effects can inevitably influence the precision of these models (Leek et al., 2010). In this research, therefore, a new modeling algorithm, pairing, and iteration were adopted to construct an irlncRNA pair model with no requirement for expression level. The algorithm strategy also spares the trouble of data standardization or consideration of technical bias across platforms, rendering it more appropriate for scientific and clinical research (Zak et al., 2016; Negi et al., 2019). The efficacy of the model was eventually confirmed by its predictive value, diagnostic effectiveness, antitumor drug efficacy, and tumor immune infiltration among patients with CRC.

## Materials and Methods

### Acquisition of COAD and Rectal Adenocarcinoma Data From the Cancer Genome Atlas

From the Genomic Data Commons of the Cancer Genome Atlas (TCGA) database, COAD, and rectal adenocarcinoma (READ) transcriptome profiling (RNA-Seq) data normalized to fragments per kilobase of transcript per million mapped reads were downloaded (Cancer Genome Atlas Network, 2012). Clinical information on patients with CRC was retrieved from the COAD and READ datasets of the TCGA. Patients were excluded from this study if (a) their follow-up time was 0 days or unknown and (b) their survival status was not clear.

### Preparation and Processing of Data

From the Ensembl website,^1^ gene transfer format (GTF) files were collected for annotation. By combining the downloaded transcriptome profiling (RNA-Seq) data and GTF files in the Perl language, we screened out the mRNAs and lncRNAs for future analysis. The demographic information as well as the survival time and status of each patient were obtained through the Perl procedure. Detailed clinical characteristics are listed in Supplementary Table 1.

### Acquisition of irlncRNAs and Differential Expression Analysis

A list of recognized immune-related genes was retrieved from the ImmPort database (Bhattacharya et al., 2014).^2^ Based on the contents of the list, immune-related genes were extracted from the downloaded data. Correlation analysis between immune-related genes and all lncRNAs obtained from the TCGA was then performed and irlncRNAs were screened. The criteria were set as follows: (1) immune gene correlation coefficient greater than 0.5 and (2) *P* value less than 0.001. After irlncRNAs were filtered, the R packages “limma (limma, version3.46.0)” and “pheatmap (pheatmap, version1.0.12)” were used for differential expression analysis and visualization. The downregulated or upregulated irlncRNAs were defined as those with a false discovery rate <0.05 and | log2-fold change| > 2, respectively.

### Pairing Differentially Expressed irlncRNAs and Construction of a 0-or-1 Matrix With IrlncRNA Pairs

The differentially expressed irlncRNAs (DE irlncRNAs) were cyclically singly paired, with the expression level of the immune-related lncRNA in any sample subject to a pairwise comparison so that a score for each irlncRNA pair was generated. The score of an irlncRNA pair was set to one if the expression level of irlncRNA A was higher than that of irlncRNA B; otherwise, the irlncRNA pair score was zero. The generated 0-or-1 matrix was then screened. The irlncRNA pair was discarded if the score of the irlncRNA pair was identical in more than 80% of all samples (Kim et al., 2016).

### Identification of Prognosis-Correlated and Immune-Related LncRNA Pairs as Well as Construction of a Risk Model to Evaluate the RiskScore

Screening by Univariate Cox regression found a total of 50 irlncRNA pairs significantly correlated with prognosis (*p* < 0.01). Then, the “glmnet (glmnet, version 4.1)” package in R software was used to conduct Lasso Cox regression analysis of the prognosis-associated irlncRNA pairs. To prevent model overfitting effects and remove these highly correlated irlncRNA pairs, a Lasso regression model with 1,000 random cycles was employed to ensure that the most informative and fewest number of irlncRNA pairs were retained in the model (Simon et al., 2011). Finally, Lasso regression analysis identified 30 irlncRNA pairs, and multivariate Cox regression analysis extracted 15 of them to set up a risk model. To determine the riskScore for patients via this risk model, the following equation was used:

$$\text{riskScore}=\sum_{i=1}^{n}CirlncRNApair_{i}\times ExpirlncRNApair_{i}$$

where CirlncRNApairi is the coefficient and ExpirlncRNApairi is the expression level of the irlncRNA pair. Through the application of the “survivalROC” package (survivalROC, version 1.0.3) and via time-dependent receiver operating characteristic (ROC) curve analysis, the optimal cutoff value for 1-year OS in the TCGA dataset was defined at the point with the highest sum of sensitivity and specificity in the ROC curve (Heagerty et al., 2000). Based on the optimal cutoff value, the patients were arranged into low- and high-risk groups, and the 1–, 3–, and 5-year ROC curves of the risk model were drawn, respectively.

### Verification and Evaluation of the Risk Model

Survival analysis was performed on both the high-risk group and the low-risk group using the log-rank test, and the results are presented as survival curves. Both univariate and multivariate Cox proportional hazards regression analyses were used to assess whether the model was qualified as an independent predictor of clinical prognosis on the TCGA cohort (*p* < 0.05 was considered statistically significant). The results are presented as a forest map. To validate the optimality of the model, we plotted and compared the 1-year ROC curves of the riskScore and other clinicopathological factors. We also used R tools to show the specific riskScore values and survival outcome of each patient in the model.

### Clinical Relevance Analysis

The correlations between the clinicopathologic characteristics and the model was analyzed by using chi-square test. A strip chart was drawn to show the results. The riskScore differences among different groups were calculated by the Wilcoxon signed-rank test based on the clinicopathologic characteristics. A box chart was used for visualization. The R packages for these analyses were “survival (survival, version3.2.7),” “survminer (survminer, version0.4.8),” “pheatmap (pheatmap, version1.0.12),” and “ggpubr (ggpubr, version0.4.0).”

### Evaluation of Tumor-Infiltrating Immune Cells

Immune infiltration status of the patients from the COAD and READ datasets in the TCGA database were calculated using the CIBERSORT, TIMER, XCELL, QUANTISEQ, MCPcounter, and EPIC algorithms. Furthermore, the Wilcoxon signed-rank test was used to analyze the differences in immune infiltrating cells between the two groups. The relationship between the riskScore and tumor-infiltrating immune cells (TIICs) was determined by Spearman correlation analysis. The results of the Wilcoxon signed-rank test and Spearman correlation analysis are shown in a box chart and a lollipop diagram, respectively. (*p* < 0.05 was considered significant.) The process was implemented using the R packages “scales (scales, version1.1.1),” “ggplot2 (ggplot2, version3.3.3),” “ggtext (ggtext, version0.1.1),” “ggpubr (ggpubr, version0.4.0),” and “limma (limma,version3.46.0).”

### Analyses of the Expression Levels of Molecules Related to Immune Checkpoints

To investigate the potential relationship between the risk model and the expression levels of molecules associated with immune checkpoints (Xu W. H. et al., 2019), we utilized the “limma (limma, version3.46.0)” and “ggpubr (ggpubr, version0.4.0)” packages to generate violin plots for visualization.

### Exploration of the Relationship Between the Risk Model and Antitumor Drugs as Well as the Relationship Between the Risk Model and Kinase Inhibitors

To assess the effect of the risk model in the clinic for CRC treatment, we investigated associations between riskScore and the effectiveness of some chemotherapeutic drugs and kinase inhibitors in treating CRC in the COAD and READ datasets of the TCGA database. Chemotherapy drugs such as bleomycin, doxorubicin, etoposide, and lenalidomide (Gandhi et al., 2013; Gamerith et al., 2014; Leuci et al., 2016) and kinase inhibitors such as IPA.3 (Khawaja et al., 2020), AZD.0530 (Arcaroli et al., 2010; Morrow et al., 2010; Reddy et al., 2015), BMS.754807 (Wang Q. et al., 2020), CCT007093 (Yin et al., 2013; Kozakai et al., 2014), OSI.906 (Flanigan et al., 2013; Leiphrakpam et al., 2014; Bendell et al., 2015), pazopanib (Arcaroli et al., 2010; Morrow et al., 2010; Reddy et al., 2015), VX.702 (Chiacchiera and Simone, 2008; Paillas et al., 2011; Hsu et al., 2012; Grossi et al., 2014; Gupta et al., 2014; Zheng et al., 2018), PD0325901 (Jing et al., 2019; Wang Q. et al., 2020), and MG.132 (Wu et al., 2008; Zhang et al., 2008) were used for CRC treatment in several studies. The Wilcoxon signed-rank test was employed to compare the differences in the half maximal inhibitory concentration (IC50) values between the two groups. The results are presented in the form of a box chart plotted by the R packages “pRRophetic (pRRophetic, version0.5)” and “ggplot2 (ggpubr, version0.4.0).”

## Results

### Identification of Differentially Expressed and Immune-Related lncRNAs

**FIGURE 1:**
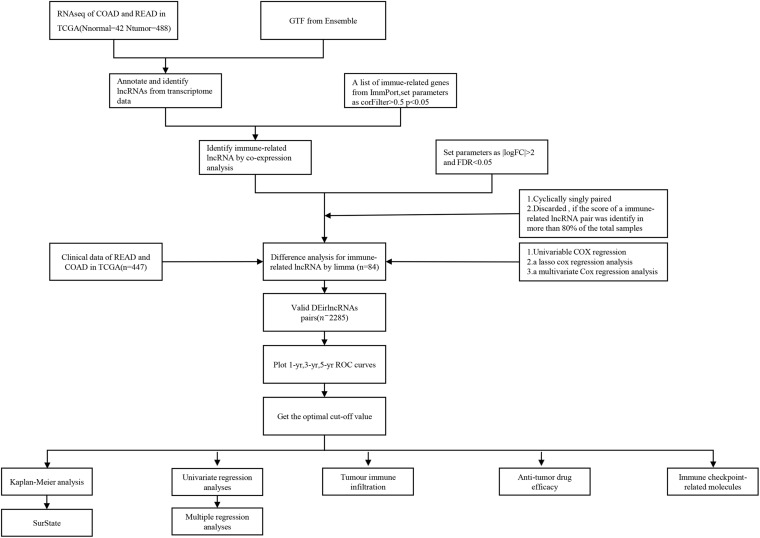
The flow diagram of the systematic study process.

**FIGURE 2:**
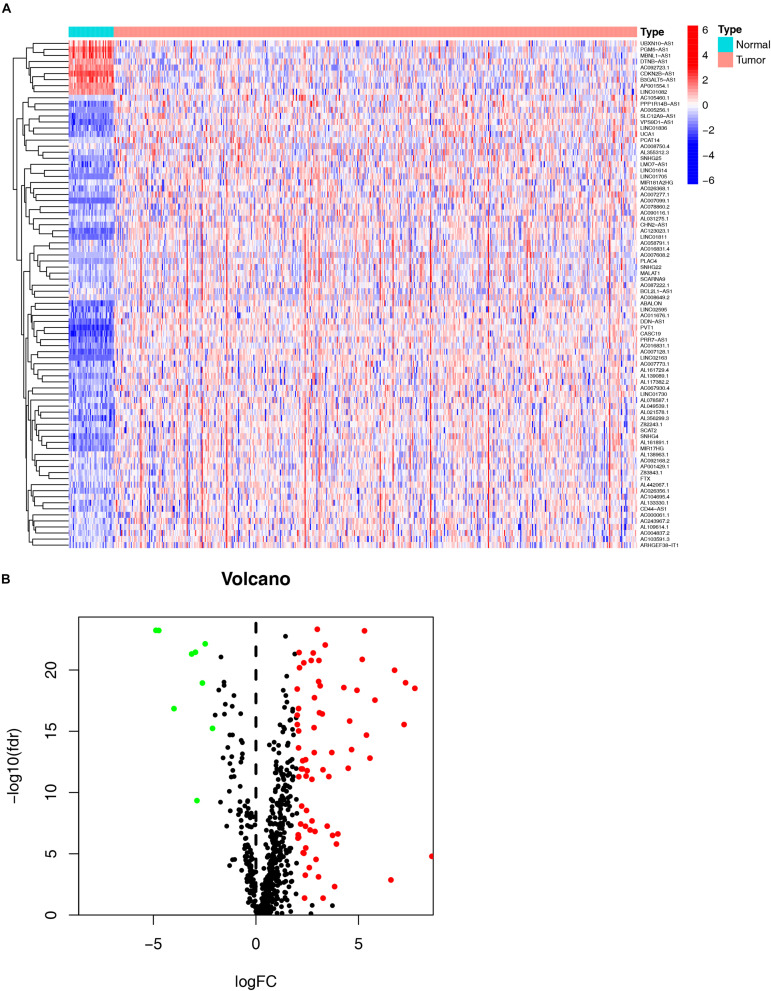
Identification of differential expression immune-related lncRNAs (DE immune-related lncRNAs). The heatmap **(A)** and volcano plot **(B)** show DE immune-related lncRNAs (DEirlncRNAs) between the colorectal cancer tumor samples and the normal samples.

As shown in the flowchart (Figure 1), the first step was to extract the transcriptome profiling data of CRC patients from the COAD and READ datasets of the TCGA database, including 42 normal and 488 tumor samples. The next step was to annotate the data based on the GTF files from Ensembl. By using the Perl language, we retrieved immune-related genes with the list from the ImmPort database. After analyzing the correlation between immune-related genes and all lncRNAs obtained from the TCGA, 654 immune-related lncRNAs were found (Supplementary Table 2). Additionally, a total of 84 DE irlncRNAs (75 upregulated and 9 downregulated) were identified between the tumor and normal samples (Figures 2A,B).

### Identification of DE irlncRNA Pairs and Construction of the Risk Model

**FIGURE 3:**
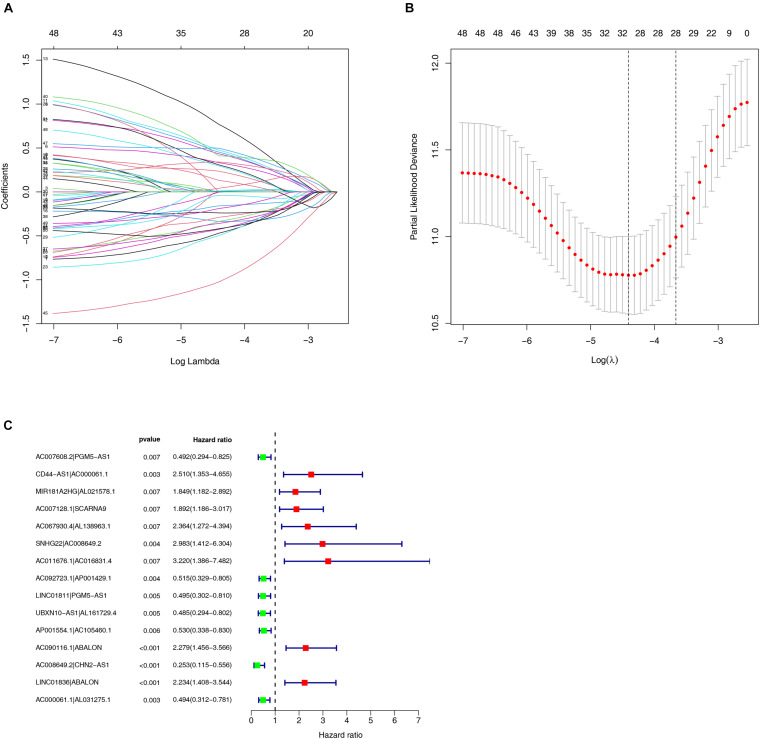
Construction of the risk model using DEirlncRNA Pairs. **(A,B)** The Lasso Cox analysis of the 30 immune-related lncRNA pairs. **(C)** A forest plot of 15 DEirlncRNA pairs selected via Cox proportional hazard regression for the construction of the risk model.

**FIGURE 4:**
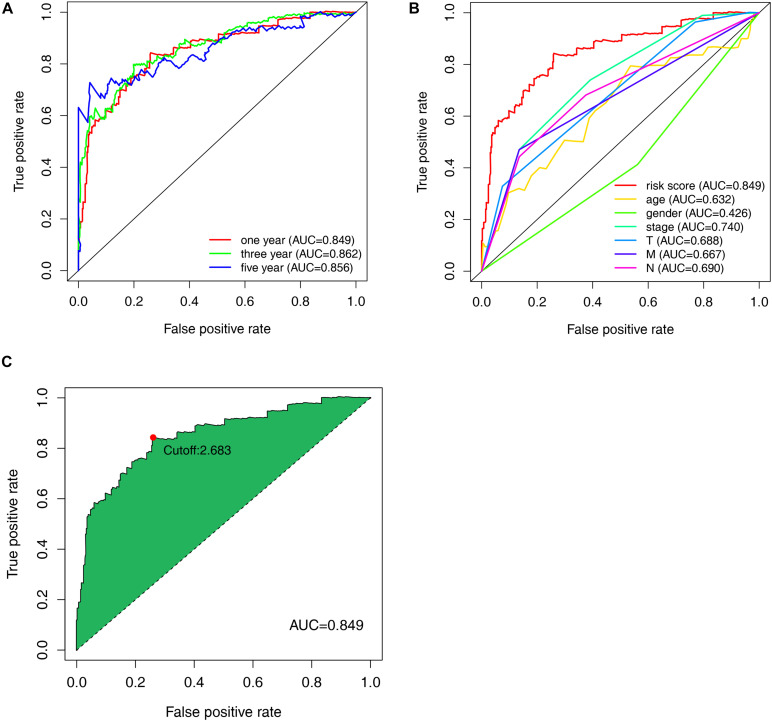
Evaluation of the constructed model through the receiver operating characteristic (ROC) curve. **(A)** Time-ROC curves of the constructed model were compared in accordance with the 1–, 3–, and 5-year ROC. The value of each area under each (ROC) curve (AUC) was over 0.85. **(B)** ROC curve analyses in predicting the overall survival (OS) of patients for risk score, age, gender, stage, T, M, and N **(C)** The optimal cut-off value was the maximum inflection point, according to the time-dependent ROC curve analysis.

Among the 84 DE irlncRNAs, 2,285 valid irlncRNA pairs were identified using the iteration loop and the 0-or-1 matrix. After the rigorous screening that removed irlncRNA pairs with comparatively small variation and the application of univariable and Lasso Cox regression analyses, 30 irlncRNA pairs were extracted (Figures 3A,B). Ultimately, the use of the stepwise method incorporated 15 of the 30 irlncRNA pairs in the Cox proportional hazards model (Figure 3C). The ROC curve was drawn, with the area under the curve calculated to confirm whether the model was reliable. The 1-, 3-, and 5-year ROC curves (Figure 4A) showed that all the areas under the curve were over 0.84. In addition, the 1-year ROC curves were compared with other clinical characteristics (Figure 4B). The optimal cutoff value was found to be at the point in the 1-year ROC curve with the highest sum of sensitivity and specificity (Figure 4C). Based on the inclusion criteria, we retrieved data from 447 acceptable CRC patients from the TCGA and determined the riskScore for them all. The patients were stratified into high- or low-risk groups for subsequent validation.

### Clinical Evaluation by the Risk Assessment Model

**FIGURE 5:**
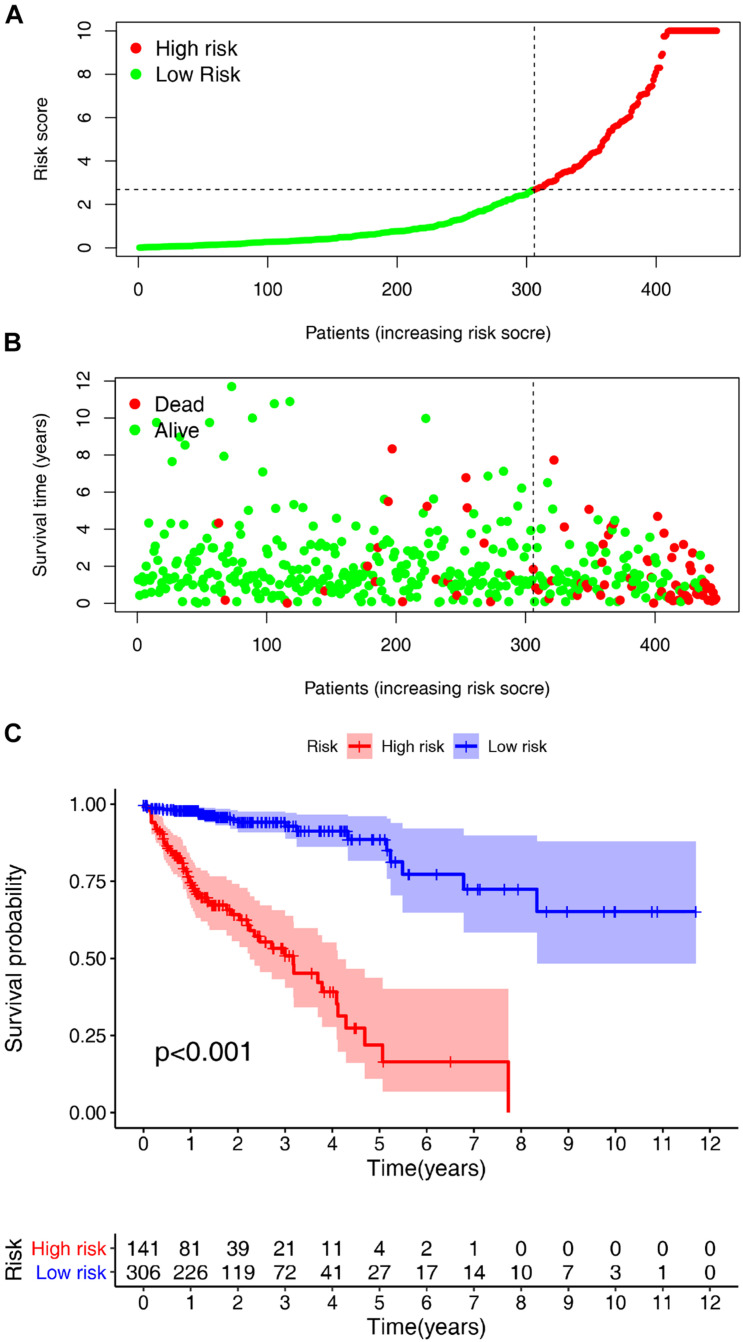
Risk score analyses and survival analyses of the constructed model in Colorectal cancer (CRC). Risk scores and survival outcome of the constructed model in CRC was plotted based on 447 patients. **(A)** Vertical coordinate represents risk scores and horizontal coordinate is the patient data. The low-risk curve was in green and the high- risk curve in red. The risk scores were increasing. **(B)** Green dots, referring to people still alive, were localized in the low-risk area; and red dots, referring to people who were already dead, were localized in the high-risk area. As the risk scores mounted, the number of deaths increased. **(C)** The survival curve indicated that the patients in the high-risk group experienced a poorer prognosis than those in the low-risk group.

**FIGURE 6:**
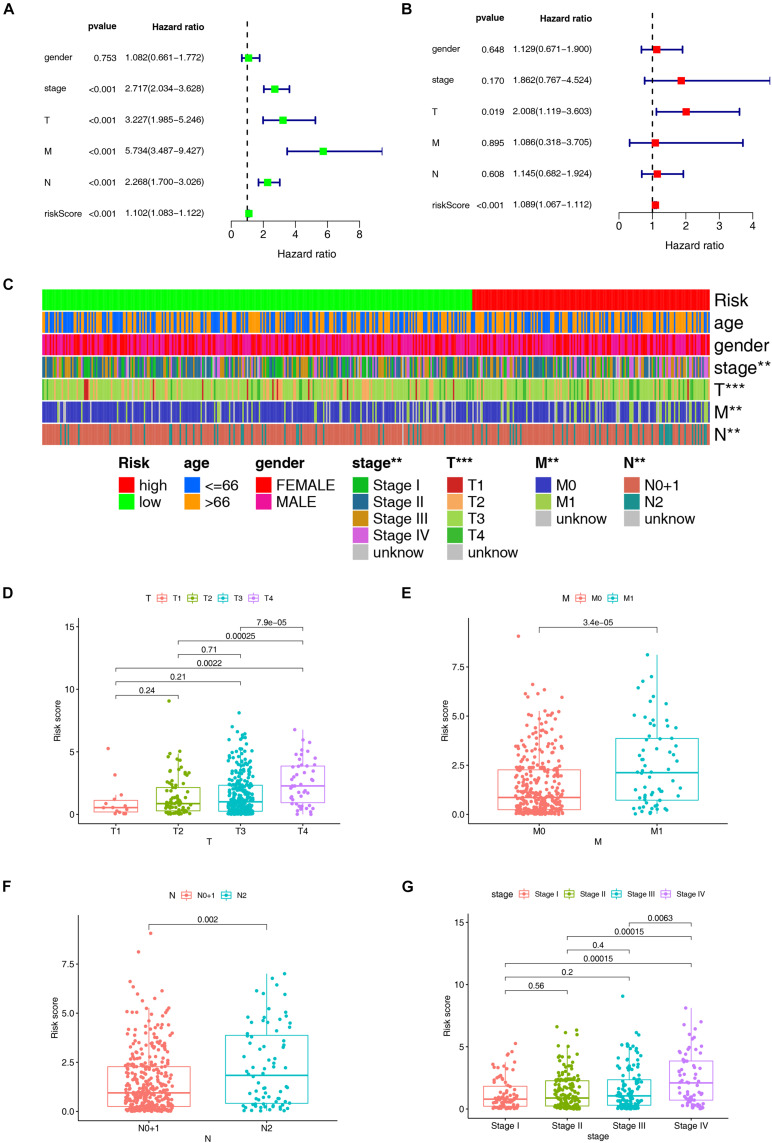
Evaluation and clinical relevance analysis of the risk model. **(A)** Forest plots of univariate Cox hazard ratio analysis revealed that pathologic tumor stage (*p* < 0.001, HR = 2.717, 95% CI [2.034–3.628]), T stage (*p* < 0.001, HR = 3.227, 95% CI [1.985–5.246]), N stage (*p* < 0.001, HR = 2.268, 95% CI [1.700–3.026]), M stage (*p* < 0.001, HR = 5.734, 95% CI [3.487–9.427]), and riskScore (*p* < 0.001, HR = 1.102, 95% CI [1.083–1.122]) were statistically different. **(B)** Forest plots of multivariate Cox hazard ratio analysis demonstrated that only T stage (p = 0.019, HR = 2.008, 95% CI [1.119–3.603]) and riskScore (*p* < 0.001, HR = 1.089, 95% CI [1.067–1.112]) served as independent prognostic predictors. A strip chart **(C)** along with the scatter diagram showed that **(D)** T stage, **(E)** M stage, **(F)** N stage, and **(G)** pathologic tumor stage were significantly connected with the riskScore. **p < 0.01; ***p < 0.001.

According to the previously confirmed optimal cutoff value, 141 patients were in the high-risk group, whereas 306 were in the low-risk group. The riskScore and survival status of each patient are shown in Figures 5A,B. Compared to the patients in the low-risk group, those in the high-risk group demonstrated significantly poorer clinical outcomes. Kaplan–Meier analysis also suggested that the patients in the high-risk group experienced a shorter survival time (*p* < 0.001) (Figure 5C). In the univariate Cox analysis, the pathologic tumor stage (*p* < 0.001, HR = 2.717, 95% CI [2.034–3.628]), T stage (*p* < 0.001, HR = 3.227, 95% CI [1.985–5.246]), N stage (*p* < 0.001, HR = 2.268, 95% CI [1.700–3.026]), M stage (*p* < 0.001, HR = 5.734, 95% CI [3.487–9.427]), and riskScore (*p* < 0.001, HR = 1.102, 95% CI [1.083–1.122]) were significantly different between the two groups according to OS (*p* < 0.01) (Figure 6A), whereas in the multivariate Cox analysis, only the T stage (*p* = 0.019, HR = 2.008, 95% CI [1.119–3.603]) and riskScore (*p* < 0.001, HR = 1.089, 95% CI [1.067–118]) were revealed as robust independent prognostic predictors (Figure 6B and Supplementary Table 3). In terms of the relationships between the clinicopathologic characteristics and the risk of CRC, the strip chart (Figure 6C) showed that T stage, M stage, N stage, and pathologic tumor stage were different in a significant manner between the high- and low-risk groups, and scatter diagrams indicated that the riskScore was significantly high in T4 stage (Figure 6D), M1 stage (Figure 6E), N2 stage (Figure 6F), and pathologic tumor stage IV (Figure 6G).

### Evaluation of Tumor-Infiltrating Immune Cells

**FIGURE 7:**
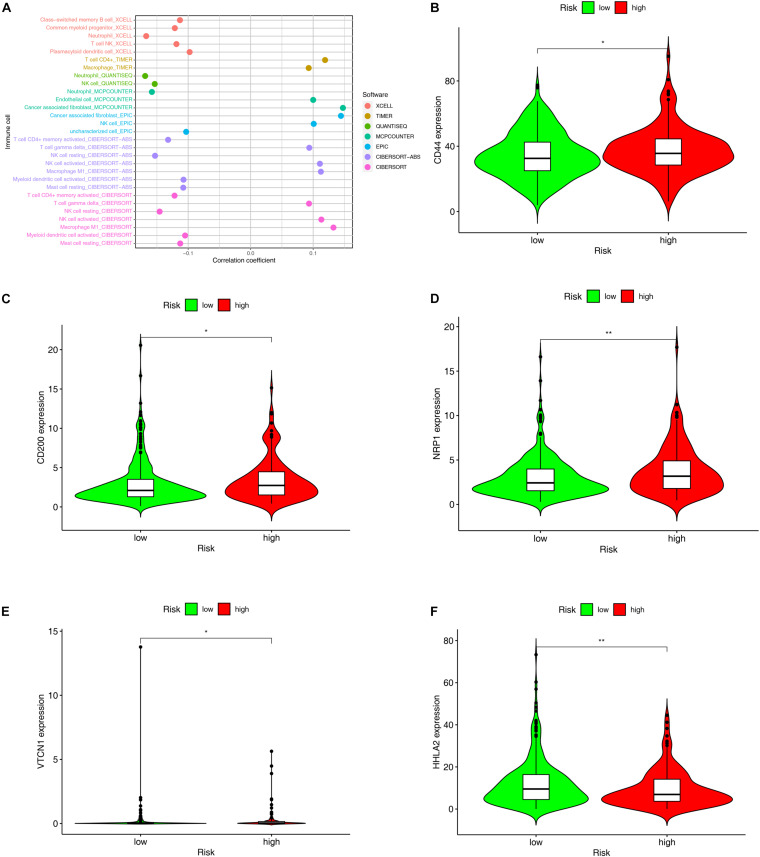
Evaluation of tumor-infiltrating immune cells (TIICs) and molecules related to immune checkpoint through the constructed model. **(A)** The Spearman correlation analysis was conducted to examine the relationship between the TIICs and the constructed model. The patients with high-risk scores in the constructed model were more positively associated with TIICs, like cancer associated fibroblast, T cell gamma delta, activated NK cell, and Macrophage M1, but negatively associated with Neutrophil, T cell CD4+ memory, NK cell resting, myeloid dendritic cells, and mast cells. **(B–F)** The expression level of molecules related to Immune Checkpoint such as **(B)** CD44, **(C)** CD200, **(D)** NRP1 were higher in the individuals with high-risk scores than those with low-risk scores, whereas the expression level of **(F)** HHLA2 was higher in the individuals with low-risk scores.*p < 0.05; **p < 0.01.

The TME is a sophisticated system comprising the extracellular matrix, chemokines, cytokines, and non-tumor cells. TIICs are non-tumor cells in the TME. Accumulating evidence suggests that what defines the behavior of cancers cells is not only the innate activities of tumor cells, but also TIICs in the TME. Through the Wilcoxon signed-rank test, we found that 13 TIICs were positively associated with the high-risk group, while 7 TIICs negatively linked with the group (Supplementary Figure 1 and Supplementary Table 4). Spearman correlation analysis revealed that 12 TIICs had a positive relation with the riskScores of the patients, whereas 17 TIICs had a negative correlation. All the results are shown in Figure 7A and Supplementary Table 5.

### Estimation of the Correlations Between the Expression Levels of Molecules Related to Immune Checkpoints and the Risk Model

Since nivolumab has been reported to provide durable responses and disease control in patients with CRC and other solid tumors (Cohen et al., 2020; Patel et al., 2021), immune checkpoint inhibitors (ICIs) have brought new hope to patients with advanced CRC. By exploring the relationships between the risk model and the expression level of the molecules related to immune checkpoints, we discovered that the expression levels of CD 44(*p* < 0.05, Figure 7B), CD200 (*p* < 0.05, Figure 7C), NRP1 (*p* < 0.01, Figure 7D), and VTCN1 (*p* < 0.05, Figure 7E) were remarkably higher in the high-risk group, while that of HHLA2 (*p* < 0.01, Figure 7F) proved to be significantly lower in this group.

### Analysis of the Correlation Between the Risk Model and Antitumor Drugs and Kinase Inhibitors

**FIGURE 8:**
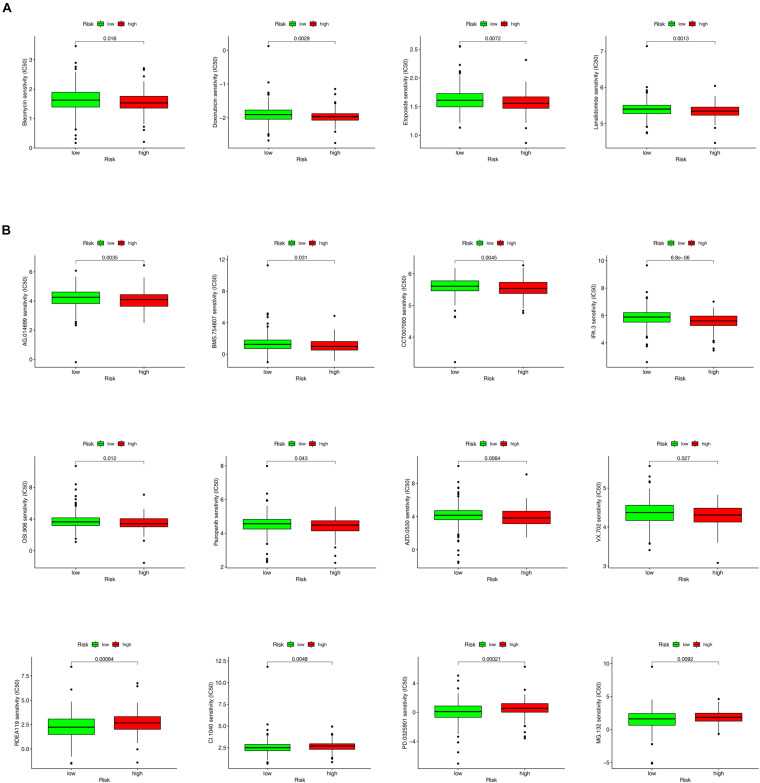
The box charts for differences in IC50 for antitumor drugs and kinase inhibitors between the high- and low-risk groups. **(A)** Chemotherapeutics such as bleomycin (*p* = 0.016), doxorubicin (*p* = 0.0029), etoposide (*p* = 0.0072), and lenalidomide (*p* = 0.0013) exhibited a lower IC50 in high-risk groups **(A)**. **(B)** Kinase inhibitors, including AG.014699 (*p* = 0.0035), BMS.754807 (*p* = 0.031), CCT007093 (*p* = 0.0045), OSI.906 (*p* = 0.012), Pazopanib (*p* = 0.043), AZD.0530 (*p* = 0.0064), VX.702 (*p* = 0.027), and IPA.3 (*p* < 0.001) showed a lower IC50 in high-risk groups. However, kinase inhibitors such as RDEA119 (*p* = 0.00064), CI.1040 (*p* = 0.0048), PD0325901 (*p* = 0.00021), MG.132 (*p* = 0.0092) showed a lower half inhibitory centration (IC50) in low-risk group **(B)**.

In addition to ICIs, several studies have reported that some chemotherapeutic drugs and inhibitors may benefit patients with CRC. The results of the Wilcoxon signed-rank test showed that the high-risk group was correlated with lower IC50 values of chemotherapeutic drugs such as bleomycin *(p* = 0.016), doxorubicin (*p* = 0.0029), etoposide (*p* = 0.0072), and lenalidomide (*p* = 0.0013) (Figure 8A) and kinase inhibitors such as IPA.3 (*p* < 0.001), AG.014699 (*p* = 0.0035), AZD.0530 (*p* = 0.0064), BMS.754807 (*p* = 0.031), CCT007093 (*p* = 0.0045), OSI.906 (*p* = 0.012), pazopanib (*p* = 0.043), and VX.702 (*p* = 0.027). However, kinase inhibitors such as CI.1040 (*p* = 0.0048), MG.132 (*p* = 0.0092), PD0325901 (*p* = 0.00021), and RDEA119 (*p* = 0.00064) showed lower IC50 values in the low-risk group (Figure 8B and Supplementary Tables 6, 7).

## Discussion

Colorectal cancer, one of the most lethal and common malignant tumors in the world, accounts for approximately 935,000 deaths in 2020. The standard treatment for CRC has been comprehensive strategies involving surgery, chemotherapy, radiation, and so on. A better understanding of CRC pathophysiology has dramatically advanced targeted therapy and immunotherapy methods used for local and advanced CRC (Ciardiello et al., 2019; Johdi and Sukor, 2020; Xie et al., 2020). Due to advances in primary and adjuvant therapies, CRC mortality rate has fallen by more than 20% over the past decade. Despite the lengthening of survival time, the mortality rate for metastatic CRC remains unacceptably high and the highest survival rate is still seen primarily among non-metastatic CRC (De Falco et al., 2020). As the disease tends to show symptoms only at an advanced stage, multiple diagnostic strategies, including biopsy, laboratory examinations, and colonoscopy that aim for early detection and mortality reduction, are being implemented. In recent years, owing to evolving sequencing technologies, the models using molecular biomarkers to diagnose at an early stage and evaluate the survival prognosis in CRC patients have reduced CRC mortality rate (Ahluwalia et al., 2021). However, on account of sampling bias and technical batch effects, the practical applicability of these models remains limited. In this study, we adopted immune-related gene pairing and introduced irlncRNA combinations that do not require data normalization across platforms, as this approach only needs to compare pairwise the expression level of the target lncRNA within one single sample (Gruss et al., 2019). We developed a new predictive model associated with the tumor immune microenvironment, which proves to be highly applicable for individual and clinical study. Several studies have confirmed that this model is reliable in predicting OS in patients with different types of cancer, for example ovarian carcinoma, early-stage non-small cell lung cancer (NSCLC), melanoma, and hepatocellular carcinoma. Li et al. (2017) established a prognostic model consisting of 25 immune-related gene pairs for non-squamous NSCLC, which was verified in various separate datasets across different platforms. Hong et al. (2020) forged a novel signature consisting of 12 irlncRNA pairs to predict prognosis for the patients with hepatocellular carcinoma and to categorize those who could reap benefits from antitumor immunotherapy. Zhang et al. (2019) developed a potent signature with 20 immune-related gene pairs to predict OS for serous ovarian carcinoma patients, shedding new light for postoperative treatment. Meng et al. (2020) defined a reliable prognosis-related model for melanoma formed from immune gene pairs and proved its efficacy in other independent datasets). In this study, we managed to construct a reliable prognostic model consisting of 15 irlncRNA pairs to predict OS for CRC patients. Some of the immune-related lncRNAs in the model, including *AC007128.1*, *AC007608.2*, *PGM5-AS1*, *MIR181A2HG*, and *SCARNA9*, were proved to have a relationship with the onset and progression of several malignancies. For example, Liu et al. (2020) found that the *lncRNA AC007128.1* was correlated with the OS of patients with esophageal cancer. Kouhsar et al. (2019) revealed that the *lncRNA AC007608.2* was a newly found candidate gene potentially related to non-muscle-invasive bladder cancer. Yang demonstrated that increases in miR-484 expression levels, which may be induced by downregulation of the *lncRNA PGM5-AS1*, prohibited the promotion, and metastasis of CRC (Shen et al., 2021). Wu et al. (2020) emphasized the value of the *lncRNA MIR181A2HG* as an indicator of prognosis and the immunotherapeutic targets in bladder cancer. Wang Z. et al., 2020 reported that an lncRNA signature that includes the *lncRNA SCARNA9* may be the potential novel therapeutic target and biomarker for human endometrial cancer. However, to our knowledge, some of the immune-related lncRNAs in the model, such as *lncRNAs CD44-AS1*, *AL021578.1*, *AC067930.4*, and *AL138963.1*, have not been reported in CRC or other kinds of cancer. Their functions and mechanisms in tumors are still a mystery, and further research on their functions in CRC or their mechanisms in the tumor immune response may reveal more novel biomarkers for CRC.

In our study, a robust prognostic model consisting of 15 irlncRNA pairs was established to predict OS for CRC patients. This novel model could be applied clinically to categorize colorectal patients according to the optimal cutoff value. After differentiating patients in the high- and low-risk groups, the survival outcome was reevaluated by the log-rank test. The prognosis of the patients in the high-risk group was significantly poorer than that of those in the low-risk group. Univariate and multivariate regression analyses indicated that the model was dramatically superior to other clinical factors, such as pathologic tumor stage, T stage, N stage, and M stage, in distinguishing patients with different levels of prognosis. We also analyzed the relationships between the clinicopathologic characteristics and the risk of CRC by the chi-square test and Wilcoxon signed-rank test. The results suggest that T stage, M stage, N stage, and pathologic tumor stage were significantly related to the risk of CRC. It is our understanding that this model can be used for a better management of CRC patients.

Tumor-infiltrating immune cells is crucial in tumor progression and treatment. The types and densities of TIICs not only have connection with prognosis of patients, but also influence responses to treatment (Zhu et al., 2020; Li et al., 2021; Xu et al., 2021; Yang B. et al., 2021; Yang Y. et al., 2021). Hence, understanding the role of TIICs may provide fresh insights into searching for novel clinical biomarkers for malignancies and facilitating cancer patient treatment by means of immunotherapy. Some TIICs, such as neutrophils, cancer-associated fibroblasts (CAFs), memory CD4+ T cells, gamma delta T cells, resting natural killer (NK) cells, activated NK cells, M1 macrophages, myeloid dendritic cells, and mast cells, have been identified to correlate with the development and progression of various malignancies, especially CRC. For example, Wikberg et al. (2017) found that the low proportion of neutrophils in the tumor front was linked to a poor prognosis in the early stages of colon cancer. Kwak et al. (2014) observed a loss of PTEN in CAFs in CRC patients, and found that the absence of PTEN expression in CAFs in distant metastases may induce poor prognosis of patients. Ye et al. (2019) noticed that CRC patients with a diminished number of tumor-associated macrophages (TAMs) and an increased number of tumor-associated neutrophils survived longer than their counterparts. Tan et al. (2005) revealed that mast cell and TAM counts was in connection with the CRC patients’ clinical outcome and that an increase in mast cells and TAMs may be beneficial to the postoperative prognosis of CRC patients. In our research, we explored the relationship between the riskScore and TIICs using seven common acceptable algorithms. The riskScore was found to be positively correlated with TIICs such as CAFs, gamma delta T cells, activated NK cells, and M1 macrophages but negatively correlated with neutrophils, memory CD4+ T cells, resting NK cells, myeloid dendritic cells, and mast cells. Nowadays, immune checkpoint inhibitors (ICI), the agents which include antibodies that target CTLA4 or PD-1/PD-L1, have brought a new era of precision medicine for the treatment of cancer patients with progressive stage (Callahan et al., 2016). Since Immunotherapy with PD-1 inhibitors have become the first-line therapy for intractable microsatellite instability (MSI)-H CRC (Oliveira et al., 2019), predictive biomarkers are urgently needed which can be used to instruct clinicians to treat patients with progressive CRC receiving immunotherapy. Tumor mutational burden (TMB), the number of somatic missense mutations per million bases (MB) in a tumor’s genes, is a novel predictive biomarker of clinical response to immune checkpoint inhibitors therapy (Merino et al., 2020). Tumors with high TMB generate many antigens, making them immunogenic, and mounting an anti-tumor response (McGranahan et al., 2016). Correlation between different TMB levels and clinical efficacy of ICI therapy has been reported in several tumors include melanoma, urothelial carcinoma, and lung cancer (Hellmann et al., 2018; Zhu et al., 2018; Arora et al., 2019; Hilke et al., 2020). In CRC, Schrock et al. (2019) has revealed that high-TMB appears to have the significant correlation with longer progression-free survival and OS according to the univariate and multivariate analysis within MSI-H mCRC patients. Lee et al. (2019) has reported that TMB-high had a tendency of favorable OS and 5-year relapse-free survival compared with TMB-low in patients with CRC treated with adjuvant fluoropyrimidine and oxaliplatin chemotherapy. Although promising, the requirement of large amounts of tissue for TMB calculating and lack of normalization have limited the clinical utility of TMP. Because plasma is available, less invasive compared with tissue biopsies, and reflects the whole tumor burden present within the patient, Many studies have been focused on new immune-related plasma biomarkers (Ding et al., 2021; Yang D. et al., 2021). Ding et al. (2021) have also investigated the relationship between different lncRNA expression patterns and TMB levels and revealed that lncRNA may act as a sorter to predict the TMB level in colon cancer patients. In our research, consistent with the role of ICIs in activating the host immune response, the high-risk group was positively correlated with molecules related to immune checkpoints such as CD44, CD200, NRP1, and VTCN1. A better knowledge of the relationship between the risk model and molecules related to immune checkpoints as well as the correlation between the Risk Model and TMB may promote the action of immunotherapy in CRC.

We also analyzed the correlation between the risk model and antitumor drugs as well as the correlation between the risk model and kinase inhibitors. It is clear that combining systemic therapies, including surgical resection, chemotherapy, kinase inhibitors, and immunotherapy, may represent an effective strategy in the treatment of CRC.

There are some limitations in our study. First, the risk model was constructed on the basis of retrospective study. Because sources of bias and confounding are more common in retrospective study, it is generally believed that retrospective study designs are inferior to prospective study designs. Second, the model constructed in this study consisted of 15 irlncRNA pairs that needed to be tested simultaneously, but sequencing technologies are expensive, thus limiting the routine application of the model in clinical practice. Third, although we constructed an irlncRNA pair model to reduce certain cross-study batch effects, some may still remain. Finally, it will be beneficial to validate the constructed model by external clinical datasets. Hence, our future work should incorporate more datasets from different platforms to validate the model, collect more clinical samples, and expand the sample sizes for further verification.

## Conclusion

In summary, the proposed irlncRNA pair-based model is a promising prognostic biomarker for CRC. This model is applicable for both scientific study and clinical practice, as it is able to minimize sample errors due to variations in expression while requiring no data standardization. Prospective studies will combine various biological processes and may provide a more thorough molecular picture of CRC.

## Data Availability Statement

The datasets presented in this study can be found in online repositories. The names of the repository/repositories and accession number(s) can be found in the article/Supplementary Material.

## Ethics Statement

We acknowledge TCGA database for providing their platforms and contributors for uploading their meaningful datasets. Written informed consent for participation was not required for this study in accordance with the national legislation and the institutional requirements.

## Author Contributions

CM and XZ performed bioinformatic analysis, wrote the manuscript, and compiled the figures. NZ and SZ collected and reprocessed the data. XuZ and YZ revised the manuscript. PL contributed to conception, design, and supervision of the project. All authors contributed to the study and approved the submitted version.

## Conflict of Interest

The authors declare that the research was conducted in the absence of any commercial or financial relationships that could be construed as a potential conflict of interest.

## Publisher’s Note

All claims expressed in this article are solely those of the authors and do not necessarily represent those of their affiliated organizations, or those of the publisher, the editors and the reviewers. Any product that may be evaluated in this article, or claim that may be made by its manufacturer, is not guaranteed or endorsed by the publisher.

## Footnotes

1
https://asia.ensemble.org

2
http://www.immport.org/

## Supplementary Material

Supplementary Figure 1 The relative proportion of tumor infiltrating immune cells between the high- and low-risk groups.
