# Supplementary figures and images for: Predicting the Survival and Immune Landscape of Colorectal Cancer Patients Using an Immune-Related lncRNA Pair Model
Source: Front Genet. 2021 Sep 6;12:690530. doi: 10.3389/fgene.2021.690530 (PMC8451271; doi:10.3389/fgene.2021.690530)

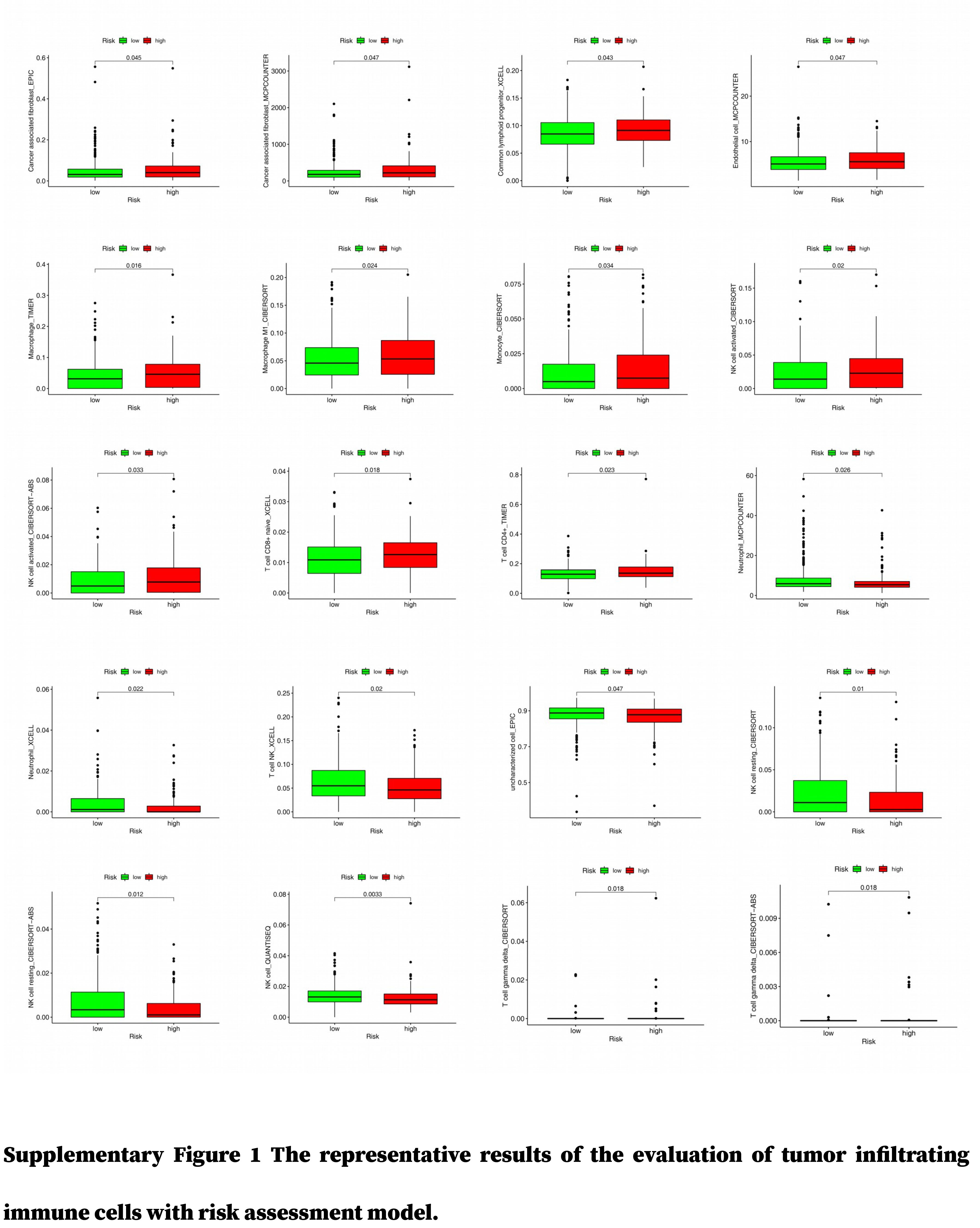

Supplement: Supplementary Figure 1 — The relative proportion of tumor infiltrating immune cells between the high- and low-risk groups. [file Image_1.TIF]
