# Supplementary material for: Predicting the Survival and Immune Landscape of Colorectal Cancer Patients Using an Immune-Related lncRNA Pair Model
Source: Front Genet. 2021 Sep 6;12:690530. doi: 10.3389/fgene.2021.690530 (PMC8451271; doi:10.3389/fgene.2021.690530)
Supplement: Supplementary file 2 [file Data_Sheet_1.docx]

| **Supplementary Table1. Clinical features of the 447 colorectal cancer patients** | | | |
| --- | --- | --- | --- |
| Characteristics | NO. | Low group | High group |
| Age(years) |  |  |  |
| $\leq66$ | 213 | 146 | 67 |
| $>66$ | 234 | 142 | 92 |
| Sex |  |  |  |
| Female | 202 | 128 | 74 |
| Male | 245 | 160 | 85 |
| N staging system |  |  |  |
| N0 | 261 | 181 | 80 |
| N1 | 109 | 69 | 40 |
| N2 | 76 | 37 | 39 |
| Unknown | 1 | 1 | 0 |
| T staging system |  |  |  |
| T1 | 14 | 12 | 2 |
| T2 | 81 | 56 | 25 |
| T3 | 305 | 201 | 104 |
| T4 | 46 | 18 | 28 |
| Unknown | 1 | 1 | 0 |
| M staging system |  |  |  |
| M0 | 335 | 228 | 107 |
| M1 | 66 | 31 | 35 |
| Unknown | 46 | 29 | 17 |
| Tumor stage |  |  |  |
| Stage I | 80 | 59 | 21 |
| Stage II | 165 | 112 | 53 |
| Stage III | 120 | 77 | 43 |
| Stage IV | 67 | 32 | 35 |
| Unknow | 15 | 8 | 7 |

Supplementary Table 2. A total of 654 immune-related lncRNAs were identified by co-expression analysis (submitted as a separate Excel file).

| **Supplementary Table 3. The detail values of univariate and multivariate cox regression analysis.** | | | | | | | | |
| --- | --- | --- | --- | --- | --- | --- | --- | --- |
| id | Univariate analysis | | | | Multivariate analysis | | | |
|  | HR | HR.95L | HR.95H | P | HR | HR.95L | HR.95H | P |
| age | 1.04 | 1.01 | 1.06 | 0.003 | 1.04 | 1.02 | 1.07 | 0.000 |
| gender | 1.08 | 0.66 | 1.77 | 0.753 | 1.13 | 0.67 | 1.90 | 0.648 |
| stage | 2.72 | 2.03 | 3.63 | 1.28e^-11^ | 1.86 | 0.77 | 4.52 | 0.170 |
| T | 3.23 | 1.99 | 5.25 | 2.28e^-06^ | 2.01 | 1.12 | 3.60 | 0.019 |
| M | 5.73 | 3.49 | 9.43 | 5.83e^-12^ | 1.09 | 0.32 | 3.71 | 0.895 |
| N | 2.27 | 1.70 | 3.03 | 2.55e^-08^ | 1.15 | 0.68 | 1.92 | 0.608 |
| riskScore | 1.10 | 1.08 | 1.12 | 5.11e^-27^ | 1.09 | 1.07 | 1.11 | 4.44e^-16^ |

| **Supplementary Table 4. The p value of comparing tumour infiltrating immune cells and riskScore** | |
| --- | --- |
| Symbol | pVal |
| Cancer associated fibroblast_EPIC | 0.045 |
| Cancer associated fibroblast_MCPCOUNTER | 0.047 |
| Common lymphoid progenitor_XCELL | 0.043 |
| Endothelial cell_MCPCOUNTER | 0.047 |
| Macrophage M1_CIBERSORT | 0.024 |
| Macrophage_TIMER | 0.016 |
| Monocyte_CIBERSORT | 0.034 |
| NK cell activated_CIBERSORT−ABS | 0.033 |
| NK cell activated_CIBERSORT | 0.020 |
| T cell CD4+_TIMER | 0.023 |
| T cell CD8+ naive_XCELL | 0.018 |
| T cell gamma delta_CIBERSORT−ABS | 0.018 |
| T cell gamma delta_CIBERSORT | 0.018 |
| Neutrophil_MCPCOUNTER | 0.026 |
| Neutrophil_XCELL | 0.022 |
| NK cell resting_CIBERSORT−ABS | 0.012 |
| NK cell resting_CIBERSORT | 0.010 |
| NK cell_QUANTISEQ | 0.003 |
| T cell NK_XCELL | 0.020 |
| uncharacterized cell_EPIC | 0.047 |

| **Supplementary Table 5. The detail comparison results of correlation ship between tumour infiltrating immune cells and riskScore.** | | |
| --- | --- | --- |
| symbol | correlation | pValue |
| T cell CD4+_TIMER | 0.119 | 0.012 |
| Macrophage_TIMER | 0.093 | 0.050 |
| T cell CD4+ memory activated_CIBERSORT | -0.122 | 0.010 |
| T cell gamma delta_CIBERSORT | 0.093 | 0.049 |
| NK cell resting_CIBERSORT | -0.146 | 0.002 |
| NK cell activated_CIBERSORT | 0.113 | 0.017 |
| Macrophage M1_CIBERSORT | 0.133 | 0.005 |
| Myeloid dendritic cell activated_CIBERSORT | -0.105 | 0.027 |
| Mast cell resting_CIBERSORT | -0.113 | 0.017 |
| T cell CD4+ memory activated_CIBERSORT-ABS | -0.132 | 0.005 |
| T cell gamma delta_CIBERSORT-ABS | 0.094 | 0.048 |
| NK cell resting_CIBERSORT-ABS | -0.153 | 0.001 |
| NK cell activated_CIBERSORT-ABS | 0.111 | 0.019 |
| Macrophage M1_CIBERSORT-ABS | 0.112 | 0.018 |
| Myeloid dendritic cell activated_CIBERSORT-ABS | -0.107 | 0.023 |
| Mast cell resting_CIBERSORT-ABS | -0.108 | 0.023 |
| Neutrophil_QUANTISEQ | -0.169 | 0.000 |
| NK cell_QUANTISEQ | -0.154 | 0.001 |
| Neutrophil_MCPCOUNTER | -0.158 | 0.001 |
| Endothelial cell_MCPCOUNTER | 0.100 | 0.035 |
| Cancer associated fibroblast_MCPCOUNTER | 0.148 | 0.002 |
| Class-switched memory B cell_XCELL | -0.113 | 0.017 |
| Common myeloid progenitor_XCELL | -0.121 | 0.010 |
| Neutrophil_XCELL | -0.167 | 0.000 |
| T cell NK_XCELL | -0.119 | 0.012 |
| Plasmacytoid dendritic cell_XCELL | -0.098 | 0.039 |
| Cancer associated fibroblast_EPIC | 0.145 | 0.002 |
| NK cell_EPIC | 0.101 | 0.033 |
| uncharacterized cell_EPIC | -0.103 | 0.029 |

| **Supplementary Table 6. The p value of comparing chemotherapeutics sensitivity and risk sore.** | |
| --- | --- |
| Drug | p-value |
| Bleomycin | 0.016 |
| Doxorubicin | 0.003 |
| Etoposide | 0.007 |
| Lenalidomide | 0.001 |

| **Supplementary Table 7. The p value of comparing Kinase Inhibitors sensitivity and risk sore.** | |
| --- | --- |
| Drug | p-value |
| AG.014699 | 0.004 |
| BMS.754807 | 0.031 |
| CCT007093 | 0.005 |
| IPA.3 | 6.8e^-06^ |
| OSI.906 | 0.012 |
| Pazopanib | 0.043 |
| AZD.0530 | 0.006 |
| VX.702 | 0.027 |
| CI.1040 | 0.005 |
| MG.132 | 0.009 |
| PD.0325901 | 0.000 |
| RDEA119 | 0.000 |
